# Health Care Providers’ Advice to Quit Smoking, National Health Interview Survey, 2000, 2005, and 2010

**DOI:** 10.5888/pcd9.110340

**Published:** 2012-07-19

**Authors:** Judy Kruger, Lauren Shaw, Jennifer Kahende, Erica Frank

**Affiliations:** Author Affiliations: Lauren Shaw, Jennifer Kahende, Centers for Disease Control and Prevention, Atlanta, Georgia; Erica Frank, University of British Columbia, Vancouver, British Columbia, Canada.

## Abstract

Although the prevalence of cigarette smoking has declined in the United States, little documentation exists to ascertain which health care providers (HCPs) promote smoking cessation. We used data from the 2000, 2005, and 2010 Cancer Control Supplement of the National Health Interview Survey to examine changes in the number of adults who received smoking cessation advice from their HCP. The percentage of smokers who received cessation advice was 53.3% in 2000, 58.9% in 2005, and 50.7% in 2010. To affect noticeably declining rates, HCPs should increase their efforts to advise smokers to quit.

## MEDSCAPE CME

Medscape, LLC is pleased to provide online continuing medical education (CME) for this journal article, allowing clinicians the opportunity to earn CME credit.

This activity has been planned and implemented in accordance with the Essential Areas and policies of the Accreditation Council for Continuing Medical Education through the joint sponsorship of Medscape, LLC and Preventing Chronic Disease. Medscape, LLC is accredited by the ACCME to provide continuing medical education for physicians. 

Medscape, LLC designates this Journal-based CME activity for a maximum of 1 **AMA PRA Category 1 Credit(s)™**. Physicians should claim only the credit commensurate with the extent of their participation in the activity.

All other clinicians completing this activity will be issued a certificate of participation. To participate in this journal CME activity: (1) review the learning objectives and author disclosures; (2) study the education content; (3) take the post-test with a 70% minimum passing score and complete the evaluation at www.medscape.org/journal/pcd (4) view/print certificate.

**Release date: August 01, 2012; Expiration date: August 01, 2013**

### Learning Objectives

Upon completion of this activity, participants will be able to:

- Describe changes in the number of adults who received smoking cessation advice from their HCPs, based on data from the 2000, 2005, and 2010 Cancer Control Supplement of the National Health Interview Survey
- Describe the association between respondents’ reported desire to quit smoking and receipt of smoking cessation advice from their HCPs
- Describe other factors associated with receipt of smoking cessation advice from HCPs

**CME EDITOR**

Camille Martin, Editor, *Preventing Chronic Disease*. Disclosure: Camille Martin has disclosed no relevant financial relationships.

**CME AUTHOR**

Laurie Barclay, MD. Freelance writer and reviewer, Medscape, LLC. Disclosure: Laurie Barclay, MD, has disclosed no relevant financial relationships

**AUTHORS AND CREDENTIALS**

Disclosures: Judy Kruger, PhD, MS; Lauren Shaw, MS; Jennifer Kahende, PhD; and Erica Frank, MD, MPH have disclosed no relevant financial relationships.

Judy Kruger, PhD, MS, Epidemiology Branch, Office on Smoking and Health, Atlanta, Georgia; Lauren Shaw, MS, Jennifer Kahende, PhD, Centers for Disease Control and Prevention, Atlanta, Georgia; Erica Frank, MD, MPH, University of British Columbia, Vancouver, British Columbia, Canada.

## Objective

Tobacco use can lead to multiple serious health conditions (1), and the US Preventive Services Task Force clinical guidelines (2) strongly recommend that health care providers (HCPs) promote tobacco use cessation by offering smoking cessation advice (3,4). Although US smoking rates have declined (5), research measuring which HCPs promote smoking cessation is limited to findings from racial/ethnic studies (6,7). The objective of this study was to investigate changes since 2000 in the percentage of adults who reported receiving smoking cessation advice from their HCP and to examine correlates of receiving advice.

## Methods

We used 3 years (2000, 2005, and 2010) of cross-sectional data from the annual National Health Interview Survey (NHIS), a continuing survey of approximately 40,000 households of civilian noninstitutionalized adults aged 18 years or older in the United States. Information about NHIS methods is available at http://www.cdc.gov/nchs/nhis/methods.htm. The NHIS survey response rate was 72.1% in 2000, 69.0% in 2005, and 60.8% in 2010.We obtained data on respondents’ demographic characteristics (sex, age, race/ethnicity, education level, poverty index ratio, health insurance type) and smoking status from the entire NHIS sample for each year. The survey queried whether respondents had ever smoked 100 or more cigarettes and currently smoked every day or some days. Those responding yes to both questions were identified as current smokers. A random selection of NHIS respondents were asked to engage in a Cancer Control Supplement in 2000, 2005, and 2010. We limited these samples to current smokers who had seen an HCP in the past 12 months. Smokers were asked, “In the past 12 months, has a medical doctor or other health professional advised you to quit smoking or quit using other kinds of tobacco?” Respondents’ desire to quit was measured by asking, “Would you like to completely quit smoking cigarettes?”

Analyses were conducted using SAS version 9.1 (SAS Institute, Inc., Cary, North Carolina) and SUDAAN version 9.0 (Research Triangle Institute, Research Triangle Park, North Carolina) to account for the complex sample design. Data were age-adjusted based on the 2000 US Census and weighted using NHIS methods (8). Descriptive statistics for receiving HCP cessation advice were examined in 2000, 2005, and 2010. Statistical significance (*P* < .001) for linear trends was determined using orthogonal polynomial contrasts. Logistic regression reporting odds ratios (ORs) and 95% confidence intervals (CIs) were computed using 2010 data to determine characteristics associated with receiving advice to quit from an HCP.

## Results

In 2000, 53.3% of smokers received cessation advice in the past year; in 2005, 58.9% received advice; and in 2010, 50.7% received advice (Figure). Among men, 48.0% received advice in 2010, 54.8% in 2005, and 50.0% in 2000 (−2.0 percentage points overall, *P* < .001). Among women, 53.6% received cessation advice in 2010, 62.8% in 2005, and 56.0% in 2000 (−2.4 percentage points overall, *P* < .001).

**Figure:**
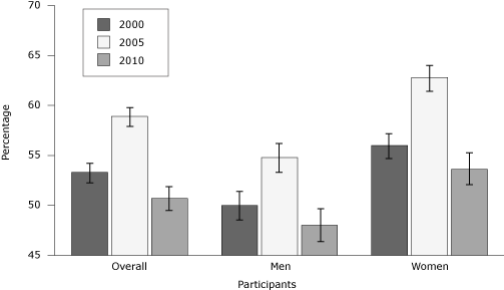
Percentage of current smokers (aged ≥18) who received health care provider advice to quit smoking in the past year, National Health Interview Survey, 2000, 2005, 2010. Error bars indicate 95% confidence intervals.

**Table:** 

Participant	Year/Year, UL					

	2000, %	2005, %	2010, %	2000, UL	2005, UL	2010, UL
**Overall**	53.3	58.9	50.7	1.0179641	0.958084	1.138661
**Men**	50.0	54.8	48.0	1.4371257	1.497006	1.666294
**Women**	56.0	62.8	53.6	1.257485	1.257485	1.588492

In 2010, women were more likely than men (OR, 1.25; 95% CI, 1.06–1.48) to receive advice from their HCP, and the likelihood of this advice increased with age (Table). Hispanic or Latino participants were less likely to receive smoking cessation advice than non-Hispanic whites (OR, 0.57; 95% CI, 0.43–0.76). Participants who had a college degree or higher were less likely to receive advice than those who had less than a high school or general education development diploma (OR, 0.63; 95% CI, 0.46–0.87). Current smokers who had government-assisted insurance (OR, 2.20; 95% CI, 1.71–2.83) or private/military insurance (OR, 1.75; 95% CI, 1.39–2.21) were more likely to receive advice to quit smoking than uninsured participants.

**Table:** Factors Associated With Receiving Health Care Provider Advice to Quit Smoking Among Current Smokers (Aged ≥18), by Selected Characteristics, National Health Interview Survey, 2010

Characteristic	Received Advice in 2010		

	N^a^	%^b^ (95% CI)	OR^c^ (95% CI)
**Overall**	3,966	50.7 (48.8–52.6)	NA
**Sex**			
Male	1,836	48.0 (45.2–50.8)	1 [Reference]
Female	2,130	53.6 (51.0–56.3)	1.25 (1.06–1.48)
**Age, y**			
18–24	404	33.2 (27.9–38.5)	1 [Reference]
25–34	811	45.8 (41.7–49.9)	1.80 (1.35–2.42)
35–44	716	48.5 (44.1–53.0)	2.03 (1.50–2.76)
45–64	1,587	57.9 (55.1–60.6)	2.73 (2.09–3.56)
≥65	448	59.3 (54.3–64.4)	2.81 (2.01–3.94)
**Race/ethnicity**			
Non-Hispanic white	2,588	52.3 (50.0–54.7)	1 [Reference]
Non-Hispanic black or African American	671	48.2 (43.5–52.9)	0.85 (0.68–1.08)
Hispanic or Latino	467	39.2 (33.7–44.7)	0.57 (0.43–0.76)
Other races, non-Hispanic^d^	154	48.1 (37.9–58.3)	0.87 (0.56–1.35)
**Education level**			
<High school or GED diploma	745	52.4 (48.4–56.4)	1 [Reference]
High school diploma	1,335	50.0 (46.5–53.5)	0.78 (0.60–1.00)
Some college	1,333	52.4 (49.3–55.5)	0.86 (0.67–1.10)
≥College degree	542	48.3 (43.1–53.4)	0.63 (0.46–0.87)
**Poverty index ratio** ^e^			
<1.25	1,717	50.1 (47.2–53.0)	1 [Reference]
1.25–3.49	1,085	52.0 (48.4–55.7)	1.12 (0.88–1.41)
≥3.50	836	54.0 (49.8–58.2)	1.20 (0.95–1.52)
**Health insurance type**			
Uninsured	880	34.0 (28.0–40.0)	1 [Reference]
Government-assisted^f^	956	56.3 (52.5–60.2)	2.20 (1.71–2.83)
Private/military	2,119	53.5 (50.8–56.2)	1.75 (1.39–2.21)

Abbreviations: CI, confidence interval; OR, odds ratio; NA, not applicable; GED, general education development diploma.

^a^ Total unweighted number of respondents.

^b^ Prevalence rates are age-adjusted to the 2000 US Census population.

^c^ ORs were adjusted for all other covariates. ORs compare the yes to no for received advice to quit in 2010.

^d^ Other refers to American Indian/Alaska Native, Asian, Native Hawaiian, or Other Pacific Islander.

^e^ Poverty index ratio was categorized as below the poverty level (<1.25), at the poverty level (1.25–3.49), and above the poverty level (≥3.50).

^f^ Refers to Medicaid, Medicare, or other public or government insurance.

In 2010, 67.7% of smokers wanted to quit. A positive correlation was found between respondents who wanted to quit smoking and those who received smoking cessation advice from their HCP. Among smokers who wanted to quit, 68.8% received cessation advice from their provider. Respondents who received advice to stop smoking from an HCP were more likely to want to quit smoking than those who did not receive such advice (OR, 1.94; 95% CI, 1.61–2.33).

## Discussion

In the United States, the number of patients reporting smoking cessation advice from HCPs initially increased from 2000 to 2005 then decreased from 2005 to 2010 to pre-2000 levels. Between 1974 and 1990, Malarcher et al found a positive trend in HCP advice to quit among both people with and without diabetes (9). Changes in the design of the Cancer Control Supplement questionnaire from 1990 to 2000 and 2005 to 2010 may explain the changes in the percentage of smokers advised by HCPs (10). Because advice from an HCP can increase quit attempts (2), findings suggest that further efforts are needed to disseminate guidelines and best practices in tobacco control to providers, such as promotion of Public Health Service clinical guidelines for treating tobacco use and dependence (3). Approximately 19.3% of adults smoked cigarettes between 2001 and 2010 (10), with smoking more prevalent among American Indian/Alaska Natives than other racial/ethnic groups (5). Similar to findings of previous studies (6,7,10), our data showed that Hispanics and Latinos were less likely to receive advice to quit than non-Hispanic whites.

We found that receiving cessation advice was strongly related to the desire to stop smoking: smokers advised by HCPs to quit were nearly twice as likely as those who did not receive such advice to want to stop smoking. Other researchers have shown a positive relationship between physician advice and patient action (11) that encourages increased cessation attempt rates. However, patients who wanted to stop smoking may have been more likely to seek or remember physician advice on the topic.

Limitations of our study include the use of self-reported data; however, NHIS uses standard questions that are well-accepted (12). Also, data were cross-sectional, which precludes demonstrating causality. Finally, a change in the order of survey questions may have resulted in discrepancies in the temporal trend. In 2010, the Cancer Control Supplement asked the question about receiving HCP advice at the end of the survey, while the question was asked in the middle of the 2005 survey.

With only half of HCPs encouraging smokers to quit and declining rates of cessation advice overall, increased efforts are essential to motivate HCPs to provide cessation advice that ultimately will yield more quit attempts and higher cessation rates.

## Figures and Tables

**Table:** 

**1. The activity supported the learning objectives.**				
**Strongly Disagree**				**Strongly Agree**
1	2	3	4	5
**2. The material was organized clearly for learning to occur.**				
**Strongly Disagree**				**Strongly Agree**
1	2	3	4	5
**3. The content learned from this activity will impact my practice.**				
**Strongly Disagree**				**Strongly Agree**
1	2	3	4	5
**4. The activity was presented objectively and free of commercial bias.**				
**Strongly Disagree**				**Strongly Agree**
1	2	3	4	5

## Post-Test Information

To obtain credit, you should first read the journal article. After reading the article, you should be able to answer the following, related, multiple-choice questions. To complete the questions (with a minimum 70% passing score) and earn continuing medical education (CME) credit, please go to http://www.medscape.org/journal/pcd. Credit cannot be obtained for tests completed on paper, although you may use the worksheet below to keep a record of your answers. You must be a registered user on Medscape.org. If you are not registered on Medscape.org, please click on the "Register" link on the right hand side of the website to register. Only one answer is correct for each question. Once you successfully answer all post-test questions you will be able to view and/or print your certificate. For questions regarding the content of this activity, contact the accredited provider, CME@medscape.net. For technical assistance, contact CME@webmd.net. American Medical Association's Physician's Recognition Award (AMA PRA) credits are accepted in the US as evidence of participation in CME activities. For further information on this award, please refer to http://www.ama-assn.org/ama/pub/category/2922.html. The AMA has determined that physicians not licensed in the US who participate in this CME activity are eligible for **AMA PRA Category 1 Credits™**. Through agreements that the AMA has made with agencies in some countries, AMA PRA credit may be acceptable as evidence of participation in CME activities. If you are not licensed in the US, please complete the questions online, print the AMA PRA CME credit certificate and present it to your national medical association for review.

## Post-Test Questions

### Article Title: Rate of Smoking Cessation Advice by HCPs Is Declining

**CME Questions**

1. You are a consultant to an HMO regarding improving smoking cessation rates among their patients. Based on an analysis of the National Health Interview Survey by Dr. Kruger and colleagues, which of the following statements about changes from 2000-2010 in the number of adults who received smoking cessation advice from their HCPs is **most likely** correct?
  - The percentage of smokers who received cessation advice improved from 2000 to 2005 and then plateaued
  - The percentage of smokers who received cessation advice was 53.3% in 2000, 58.9% in 2005, and 50.7% in 2010
  - The percentage of male smokers who received cessation advice improved from 2000 to 2010
  - The percentage of female smokers who received cessation advice improved from 2005 to 2010
2. Based on an analysis of the National Health Interview Survey by Dr. Kruger and colleagues, which of the following statements about the association between respondents’ reported desire to quit smoking and receipt of smoking cessation advice from their HCP is **most likely** correct?
  - Among smokers who wanted to quit, 68.8% received cessation advice from their provider
  - Smokers advised by HCPs to quit were about 30% more likely to want to stop smoking than those who did not receive such advice
  - The study proves that advice from HCPs to quit smoking makes smokers want to quit
  - There was no correlation between wanting to quit smoking and receiving smoking cessation advice from the HCP
3. Based on an analysis of 2010 data from the National Health Interview Survey by Dr. Kruger and colleagues, which of the following factors would **most likely** be associated with patients’ receipt of smoking cessation advice from their HCPs?
  - Male sex
  - Younger age
  - Hispanic ethnicity
  - Government-assisted or private/military insurance

**Evaluation**
